# Pre-hospital triage performance and emergency medical services nurse’s field assessment in an unselected patient population attended to by the emergency medical services: a prospective observational study

**DOI:** 10.1186/s13049-020-00766-1

**Published:** 2020-08-17

**Authors:** Carl Magnusson, Johan Herlitz, Christer Axelsson

**Affiliations:** 1 Department of Molecular and Clinical Medicine, Institute of Medicine, Sahlgrenska Academy, University of Gothenburg, Gothenburg, Sweden; 2 Pre Hospen-Centre for Prehospital Research, Faculty of Caring Science, Work Life and Social Welfare, University of Borås, Borås, Sweden

**Keywords:** Triage, Emergency medical services, Pre-hospital emergency nurse, Field assessment, Patient safety

## Abstract

**Background:**

In Sweden, the rapid emergency triage and treatment system (RETTS-A) is used in the pre-hospital setting. With RETTS-A, patients triaged to the lowest level could safely be referred to a lower level of care. The national early warning score (NEWS) has also shown promising results internationally. However, a knowledge gap in optimal triage in the pre-hospital setting persists. This study aimed to evaluate RETTS-A performance, compare RETTS-A with NEWS and NEWS 2, and evaluate the emergency medical service (EMS) nurse’s field assessment with the physician’s final hospital diagnosis.

**Methods:**

A prospective, observational study including patients (≥16 years old) transported to hospital by the Gothenburg EMS in 2016. Three comparisons were made: 1) Combined RETTS-A levels orange and red (high acuity) compared to a predefined reference emergency, 2) RETTS-A high acuity compared to NEWS and NEWS 2 score ≥ 5, and 3) Classification of pre-hospital nurse’s field assessment compared to hospital physician’s diagnosis. Outcomes of the time-sensitive conditions, mortality and hospitalisation were examined. The statistical tests included Mann–Whitney U test and Fisher’s exact test, and several binary classification tests were determined.

**Results:**

Overall, 4465 patients were included (median age 69 years; 52% women). High acuity RETTS-A triage showed a sensitivity of 81% in prediction of the reference patient with a specificity of 64%. Sensitivity in detecting a time-sensitive condition was highest with RETTS-A (73%), compared with NEWS (37%) and NEWS 2 (35%), and specificity was highest with NEWS 2 (83%) when compared with RETTS-A (54%). The negative predictive value was higher in RETTS-A (94%) compared to NEWS (91%) and NEWS 2 (92%). Eleven per cent of the final diagnoses were classified as time-sensitive while the nurse’s field assessment was appropriate in 84% of these cases.

**Conclusions:**

In the pre-hospital triage of EMS patients, RETTS-A showed sensitivity that was twice as high as that of both NEWS and NEWS 2 in detecting time-sensitive conditions, at the expense of lower specificity. However, the proportion of correctly classified low risk triaged patients (green/yellow) was higher in RETTS-A. The nurse’s field assessment of time-sensitive conditions was appropriate in the majority of cases.

## Background

Triage is commonly performed at most emergency departments (ED) in order to stratify the patients based on the severity of their conditions, when the demand exceeds the available resources [1]. The triage process, regarded as the first step in medical screening, is aimed at caring first for the patients with the most critical condition [2]. With the introduction of registered nurses (RN) trained in pre-hospital triage, in the emergency medical services (EMS) in Sweden, the EMS nurses are tasked to decide on the level of care or on whether to bypass the ED for specific patient groups with myocardial infarction (MI), stroke or sepsis, for example. The EMS in Gothenburg, Sweden uses the same triage system, (RETTS-A the five-level rapid emergency triage and treatment system for adults, [RETTS-A]) as the ED, in order to start the triage process at an earlier stage and to support the EMS nurse in the decision-making process. The Swedish RETTS-A has been evaluated at the ED, where it was used to discriminate between the severity of patients’ conditions on admission and in-hospital mortality and is regarded as a reliable triage method [3]. However, it revealed lower accuracy for mortality in the elderly than in younger patients [4]. There is a lack of evidence relating to the triage systems used for all patient presentations in the EMS [5], and only a few studies have evaluated more complex triage systems. In a previous Swedish pre-hospital study of RETTS-A with non-urgent conditions triaged to the lowest level, these patients remained at a lower level of care after consultation with a general practitioner; indicating that it is possible to reduce transportation to the ED [6]. A study from Taiwan reported a better performance for predicting hospitalisation and medical resource consumption with a five-level pre-hospital triage, compared with a two-level triage conducted by emergency medical technicians (EMT) [7]. Moreover, there is evidence to support the five-level systems instead of the lower level systems [2]. In an ED in Norway, RETTS-A displayed a superior performance in detecting sepsis than the quick sepsis-related organ failure assessment (qSOFA) score [8]. Another system based on vital signs (VS), that has attracted interest in the EMS, is the national early warning score (NEWS). Both NEWS and its latest version, NEWS 2, have been studied in the pre-hospital settings. High NEWS scores are associated with adverse outcomes and predict the risk of early death [9–13]. NEWS has also performed better in hospital studies comparing several other early warning scores for short-term mortality, cardiac arrest and the need for intensive care [14]. However, the performance of RETTS-A in a pre-hospital setting in an unselected patient population is unknown. This study aimed to 1) evaluate the performance of RETTS-A, 2) compare the performance of RETTS-A with that of NEWS and NEWS 2, and, finally, 3) evaluate the EMS nurse’s field assessment with the physician’s final hospital diagnosis.

## Methods

### Design

This was a prospective observational study with a retrospective analysis of patients ≥16 years of age who were assessed by the EMS nurse at the scene, triaged according to RETTS-A, and transported to the hospital. Prospectively, we informed and conducted repetitive training with the EMS staff on RETTS-A triage, at all the nine ambulance stations in the Gothenburg EMS before the study started.

### Setting

The study was carried out in the Gothenburg EMS area, Sweden. This is an urban setting with short transportation times and three hospitals, including one level-1 trauma unit within the catchment area. The EMS covers an area of approximately 900 km2 and is inhabited by 660,000 persons (study year). Annually, the EMS assignments exceed 80,000 and, of these, 58,575 assignments are defined as primary missions with an initial patient assessment. The EMS study organisation operates with a differentiated fleet consisting of 22 units, including 18 advanced lifesaving (ALS) ambulance types, two single responders, one physician-manned unit and one scene-commanding unit. According to Sweden legislation, at least one RN is required to staff the ambulance. The majority of all RNs in the study organisation have a postgraduate education specialising in pre-hospital emergency care. When a patient dials the Swedish emergency number (112), the operator assesses the patient according to a dispatch medical index (DMI) and dispatches a unit with a priority depending on the assessed level of severity. Priority 1 is considered time-sensitive and a unit responds with lights and sirens, priority 2 is acute but not assessed as a time-sensitive condition while priority 3 comprises non-urgent assignments. Priority 4 means assignments where there is no need for medical treatment or monitoring. ALS ambulances responding to priority 1–3 and priority 4 cases are carried out by transportation vehicles staffed by EMTs.

### Study population

**Fig. 1:**
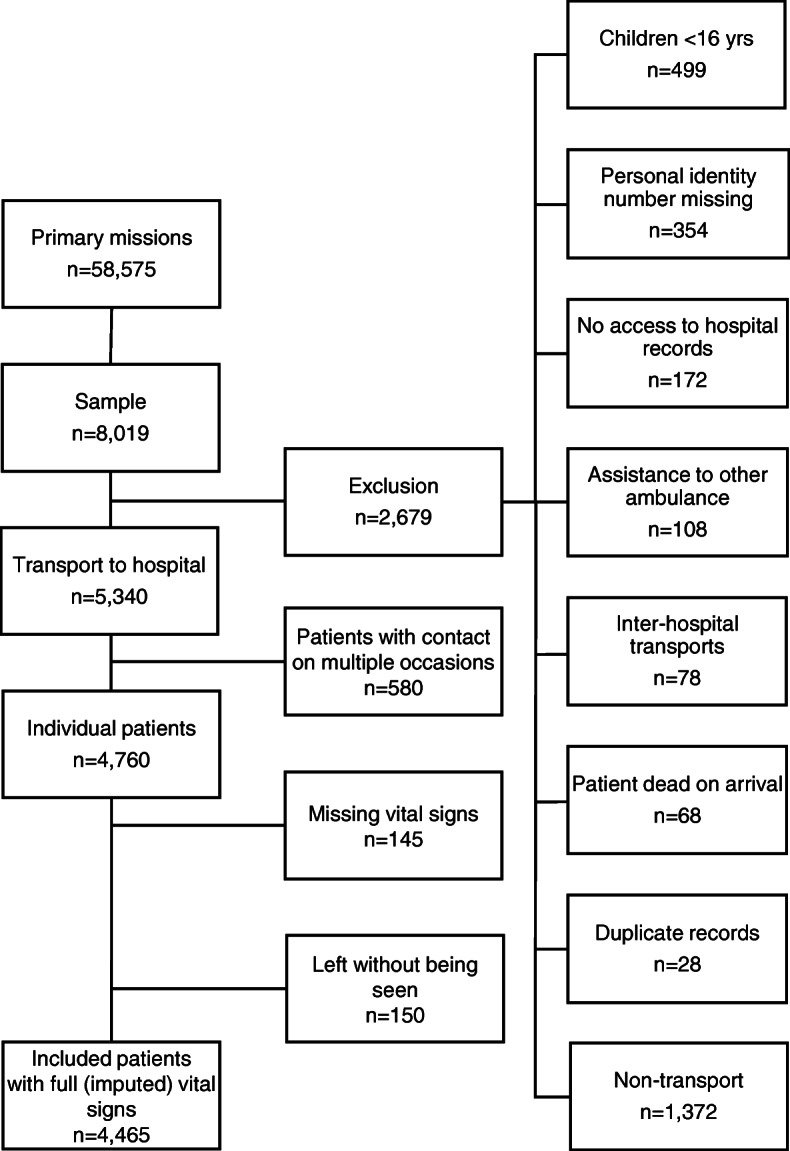
Flow chart of the studied patients

Monthly, data on the first thousand patients in a 1-year period (2016) were collected as a consecutive convenient sample. Patients were eligible for inclusion if they dialed the emergency number (112) and a unit was dispatched to the scene. The inclusion criteria were: 1) eligible for adult ED (≥16 years old) and 2) assessed at the scene by the EMS nurse and triaged according to RETTS-A. The exclusion criteria were: 1) non-conveyed patients, 2) inter-hospital transportations, 3) assistance to other ambulances, and 4) assignments with no patient contact. A total of 8019 patients were reviewed manually and, of these, 5340 adult patients were initially assessed as needing to be sent to the hospital. Patients with contact on multiple occasions were randomly excluded, making a total number of 4760 individual patients. When calculating NEWS and NEWS 2 scores, which were calculated retrospectively, another 145 patients with between four to all six VS missing, were excluded. For the remainder, if three or less VS missing (respiratory rate 1,7%; oxygen saturation 0,5%; heart rate 0,2%; systolic blood pressure 1,8%; body temperature 9,6%; level of consciousness 0,3%), they were regarded as being mostly at random (MAR) and multivariate imputation via chained equations (MICE) was performed. Another 150 patients that fulfilled all the inclusion criteria but left the ED before being seen or against medical advice and the outcome for these patients could therefore not be evaluated were excluded. Overall, 4465 unique patients were transported to the hospital with full (imputed) records of VS included (Fig. 1). The retrospective data were retrieved from EMS and hospital medical records. Each record was reviewed manually, and anonymised data such as VS, triage level, EMS nurse’s assessment and diagnosis code were then entered into a registry for calculation and statistical analysis.

### RETTS-A triage system

RETTS-A is a five-level triage system that was developed in the ED at Sahlgrenska University Hospital, Gothenburg, Sweden, in 2005 and was introduced in the EMS in 2010. RETTS-A is maintained, further developed and licensed by a Swedish company (Predicare AB) and consists of 53 flowcharts of the most common ED presentations with annual updates. Each flowchart comprises several emergency signs and symptoms (ESS), which yield a triage colour based on the severity, in combination with the VS, which are recorded in all presentations. RETTS-A colours represent the following levels of severity: red (life-threatening) and orange (potentially life-threatening), which are both defined as acute processes directly; and yellow and green, which both mean ‘can wait’ although yellow is considered to require greater urgency than green. Patients triaged to the lowest level (blue) can be managed at a lower level of care than at the ED. At the time of the study, only levels red to green were used in the EMS organisation.

### Definition of time-sensitive condition, complications and the reference patient

**Fig. 2:**
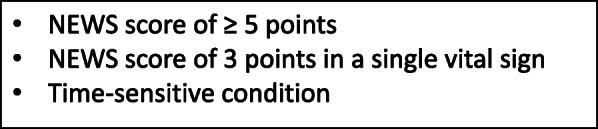
Definition of the reference patient

A time-sensitive condition is said to occur when a patient has a final diagnosis of, for example, stroke, MI or septic shock, and these conditions require prompt pre-hospital management and limited waiting time at the hospital (Additional file 1). The occurrence of complications was measured from the time of the EMS nurse’s assessment up to 48 h in the hospital. Complication was defined as any of the following occurrences: death, cardiac arrest, ventricular arrhythmias, status epilepticus, severe heart failure, hypotension, syncope and unconsciousness. It also includes deviating VS such as respiratory rate > 30 or < 8/min; oxygen saturation < 90%; heart rate > 130 rate/min (regular) or > 150 rate/min (irregular); systolic blood pressure of < 90 mmHg; body temperature > 41 °C. The reference patient in this study, indicating an ‘emergent’ patient was defined as a deviating VS in accordance with a NEWS score of ≥5; a NEWS score of 3 in a single VS; or a time-sensitive condition. The RETTS-A orange or red group (acute process directly) was then compared with the reference patient, and either of the triage levels would qualify as a true positive (Fig. 2).

### Outcomes compared with RETTS-A, NEWS, and NEWS 2

**Fig. 3:**
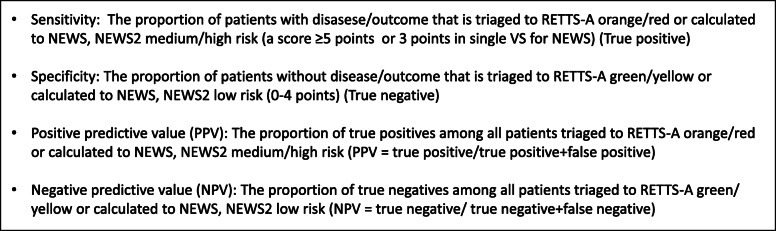
RETTS-A, NEWS and NEWS 2 categorisation in accordance with sensitivity, specificity and predictive values

Several outcome measurements have been used to compare RETTS-A, NEWS, and NEWS 2 including time-sensitive condition, occurrence of complications within 48 h, admission to in-patient care, 48-h mortality and 30-day mortality (Fig. 3). The RETTS-A and NEWS definitions of an acute patient were used to compare systems as follows: RETTS-A, orange/red level; NEWS, a score of 5 and above or a score of 3 in a single VS; NEWS 2, a score of 5 and above. In NEWS 2, only the standard scale for saturation was used, as recommended [15].

### EMS nurse’s field assessment compared with the final hospital diagnosis

In the assessment of the patient, in addition to the triage level, the EMS nurse registers the pre-hospital-assessed condition or symptoms. To categorise the EMS nurse’s assessment and relate it to the final hospital diagnosis, a classification instrument was used. The classification instrument comprises five different categories (A–E), which are related to the final diagnosis. Category A, B, C, and D correspond to a disease defined as: a time-sensitive diagnosis (stroke); a non time-sensitive diagnosis (bronchitis); a final diagnosis expressed as a symptom (non-specified chest pain); and a non-specified final diagnosis (asthenia). Only categories A–D were used, as category E only includes non-conveyed patients [16, 17]. When there was difficulty in classifying a case, it was discussed in a group consisting of a physician and senior researchers.

### Statistical analysis

The results in the study are presented as numbers, percentages or the median with percentiles (25th, 75th). In Table 1, results of group comparisons are shown by Fisher’s exact test for binary variables and the Mann–Whitney U test for continuous/ordinal variables. In Table 2, the results are presented as absolute risk (AR) and relative risk (RR). The numbers in Tables 2, 3, and 4 are presented with the corresponding confidence intervals (CIs), which has been bootstrapped 10,000 times. In Table 3, age stratification was determined by the median age of all patients in Sweden, in contact with the EMS (national pre-hospital reports, 2019). When calculating sensitivity, specificity, positive predicted value (PPV), negative predictive value (NPV), likelihood ratios and area under the receiver operating characteristic curve (AUROC) in Tables 3 and 4; RETTS-A levels (orange and red) were combined to form a 2 × 2 table and regarded as a positive test when matching the predefined reference patient. In Table 3, under-triage and over-triage were defined as 1-Sensitivity (proportion of yellow/green triaged patients among all the ‘true emergencies’) and 1-Specificity (proportion of orange/red triaged patients among all the defined ‘non-emergencies’). In Table 4, the NPV was near 1.0, and the Clopper–Pearson CI was used instead. All tests are two-sided and *p*-values < 0.01 with the 99% CI are considered significant due to the number of tests. R Studio version 1.1.463 (RStudio Inc., Boston, MA) was used to perform the data processing and the statistical analysis.

**Table 1:** Patient characteristics, EMS assessment and hospital assessment for patients transported to hospital

	Total	Red	Orange	Yellow	Green	*P*^*1*^
	*n* = 4465	*n* = 596	*n* = 1588	*n* = 1919	*n* = 362	
**Age – years (25th, 75th percentiles)**						
Median	69 (45,83)	71 (47,83)	66 (42,81)	71 (47,84)	71 (48,85)	< 0.001
**Sex – n (%)**						< 0.001
Female	2319 (51.9)	278 (46.6)	783 (49.3)	1040 (54.2)	218 (60.2)	
**Dispatcher priority – n (%) (2,5,9,2)**^**2**^						< 0.001
Priority 1	2218 (49.9)	455 (76.6)	926 (58.5)	752 (39.4)	85 (23.6)	
Priority 2	2059 (46.3)	134 (22.6)	628 (39.7)	1067 (55.9)	230 (63.9)	
Priority 3	170 (3.8)	5 (0.8)	29 (1.8)	91 (4.8)	45 (12.5)	
**Dispatch medical index**^**3**^ **- n (%) (3,7,9,3)**^**2**^						
Chest pain/cardiac disease	651 (14.7)	94 (15.9)	250 (15.8)	273 (14.3)	34 (9.5)	0.034
Extremity/wound/minor trauma	609 (13.7)	12 (2.0)	191 (12.1)	337 (17.6)	69 (19.2)	< 0.001
Abdominal/urinary tract symptoms	519 (11.7)	40 (6.7)	135 (8.5)	312 (16.3)	32 (8.9)	< 0.001
Uncertain information/suspicion of severe illness	476 (10.7)	47 (7.9)	168 (10.6)	210 (11.0)	51 (14.2)	0.089
Respiratory difficulties	462 (10.4)	152 (25.6)	137 (8.7)	136 (7.1)	37 (10.3)	< 0.001
**Time of day – n (%)**						0.001
08:00–16:00	2092 (46.9)	274 (46.0)	686 (43.2)	930 (48.5)	202 (55.8)	
16:00–24:00	1547 (34.6)	199 (33.4)	606 (38.2)	642 (33.5)	100 (27.6)	
24:00–08:00	826 (18.5)	123 (20.6)	296 (18.6)	347 (18.1)	60 (16.6)	
**Time on scene – min (25th, 75th percentile)**						
Median	22 (15,30)	25 (18,33)	22 (15,30)	21 (14,29)	21 (14,31)	< 0.001
**Medical history**^**4**^ **– n(%)**						
Diseases of the circulatory system I00-I99	4459 (30.2)	715 (33.1)	1593 (30.0)	1846 (29.8)	305 (27.9)	0.008
Mental and behavioural disorders F01-F99	2222 (15.1)	333 (15.4)	873 (16.4)	861 (13.9)	155 (14.2)	0.002
Endocrine, nutritional and metabolic diseases E00-E89	1338 (9.1)	198 (9.2)	447 (8.4)	594 (9.6)	99 (9.1)	0.186
Diseases of the musculoskeletal system and connective tissue M00-M99	1014 (6.9)	101 (4.7)	348 (6.6)	457 (7.4)	108 (9.9)	< 0.001
Diseases of the digestive system K00-K95	878 (6.0)	108 (5.0)	299 (5.6)	415 (6.7)	56 (5.1)	0.008
No medical history	569 (12.7)	59 (9.9)	211 (13.3)	249 (13.0)	50 (13.8)	0.455
**Prehospital field assessment according to RETTS-A**^**5**^ **– n (%)**						
Abdominal/flank pain	460 (10.3)	36 (6.0)	117 (7.4)	289 (15.1)	18 (5.0)	< 0.001
Chest/thoracic pain	456 (10.2)	65 (10.9)	189 (11.9)	193 (10.1)	9 (2.5)	0.003
Respiratory distress/dyspnoa/breathing difficulties	382 (8.6)	129 (21.6)	117 (7.4)	103 (5.4)	33 (9.1)	< 0.001
Unspecific condition	309 (6.9)	20 (3.4)	42 (2.6)	192 (10.0)	55 (15.2)	< 0.001
Injury head trauma	274 (6.1)	9 (1.5)	142 (8.9)	105 (5.5)	18 (5.0)	0.039
**Prehospital medication – n (%)**						
Any medication	1565 (35.1)	399 (66.9)	653 (41.1)	472 (24.6)	41 (11.3)	< 0.001
Intravenous medication	889 (19.9)	227 (38.1)	367 (23.1)	279 (14.5)	16 (4.4)	< 0.001
**Management ED – n (%)**^**6**^						
Admitted to in-patient care	2287 (51.2)	483 (81.0)	866 (54.5)	826 (43.0)	112 (30.9)	< 0.001
Extended examination – intervention ⟹ X-ray, CT, US, MR, LP	687 (15.4)	26 (4.4)	300 (18.9)	305 (15.9)	56 (15.5)	0.405
Lab, drug administration, prescription	1227 (27.5)	80 (13.4)	364 (22.9)	657 (34.2)	126 (34.8)	< 0.001
Clinical exam observation only	219 (4.9)	7 (1.2)	45 (2.8)	112 (5.8)	55 (15.2)	< 0.001
Patient managed by ED nurse, referral to primary care	45 (1.0)	0 (0.0)	13 (0.8)	19 (1.0)	13 (3.6)	0.007
Under the influence of substances (alcohol, drugs) – n (%)	398 (8.9)	67 (11.2)	216 (13.6)	91 (4.7)	24 (6.6)	< 0.001
**Days of in-patient care – n**						
Mean (SD)	7.97 (9.15)	8.53 (10.35)	7.48 (8.92)	8.06 (8.76)	8.74 (8.00)	0.463
Median (25th, 75th percentile)	5 (2,11)	6 (3,10)	5 (2,10)	6 (3,11)	6 (3,13)	0.042
**Final hospital assessment, ICD-10 codes – n (%) (8,59,62,24)**^**2**^						
(R) Symptoms, signs and abnormal clinical and laboratory findings	851 (19.7)	57 (9.7)	305 (19.9)	436 (23.5)	53 (15.7)	< 0.001
(S,T) Injury, poisoning and certain other consequnces of external causes	828 (19.2)	53 (9.0)	333 (21.8)	379 (20.4)	63 (18.6)	0.113
(I) Diseases of the circulatory system	550 (12.8)	148 (25.2)	196 (12.8)	178 (9.6)	28 (8.3)	< 0.001
(J) Diseases of the respiratory system	351 (8.1)	115 (19.6)	133 (8.7)	94 (5.1)	9 (2.7)	< 0.001
(F) Mental, behavioural and neurodevelopmental disorders	301 (7.0)	53 (9.0)	110 (7.2)	100 (5.4)	38 (11.2)	0.073
**All-cause mortality – n (%)**						
≤ 7 days	88 (2.0)	39 (6.5)	33 (2.1)	16 (0.8)	0 (0.0)	< 0.001
≤ 30 days	209 (4.7)	73 (12.2)	80 (5.0)	50 (2.6)	6 (1.7)	< 0.001
≤ 365 days	705 (15.8)	155 (26.0)	237 (14.9)	264 (13.8)	49 (13.5)	< 0.001

^1^ *P*-values calculated on red/orange and yellow/green groups respectively

^2^ Missing patients in each triage colour category respectively

^3^ The five most common dispatcher assessments according to dispatch medical index

^4^ Past medical history – one patient can have multiple diagnoses within the same category

^5^ The five most common prehospital assessments according to RETTS-A

^6^ One patient per category, descending order. *CT* computed tomography, *US* ultrasound, *MR* magnetic resonance, *LP* lumbar puncture

**Table 2:** Risk prediction of outcomes in EMS RETTS-A triage levels

		High acuity		Low acuity	
		Red	Orange	Yellow	Green
	nt	596	1588	1919	362
Time-sensitive condition	n	153	193	116	11
	AR	25.7	12.2	6.04	3.01
	RR	3.10	1.25	0.43	0.27
	CI	[2.44,3.89]	[0.99,1.57]	[0.33,0.56]	[0.09,0.50]
Deviating VS/complication within 48 h	n	303	110	39	3
	AR	50.8	6.92	2.03	0.83
	RR	12.94	0.58	0.12	0.08
	CI	[10.5,16.5]	[0.43,0.75]	[0.08,0.18]	[0.02,0.33]
Admission to in-patient care	n	483	866	826	112
	AR	81.0	54.5	43.0	30.9
	RR	1.74	1.10	0.75	0.58
	CI	[1.62,1.86]	[1.02,1.19]	[0.69,0.81]	[0.47,0.71]
48 h mortality	n	22	18	4	0
	AR	3.69	1.13	0.21	0
	RR	6.49	1.25	0.13	0
	CI	[2.93,14.75]	[0.52,2.81]	[0.03,0.50]	[0,0]
30-day mortality	n	73	80	50	6
	AR	12.8	5.04	2.6	1.66
	RR	3.48	1.12	0.42	0.34
	CI	[2.41,4.88]	[0.78,1.60]	[0.27,0.62]	[0.05,0.74]

*nt* Total number of patients per triage level, *n* number of patients, *AR* absolute risk %, *RR* relative risk, *CI* Bootstrapped 99% confidence intervals

**Table 3:** Prehospital triage according to RETTS-A compared with a reference patient

	All	Female	Male	≤ 65 yrs	> 65 yrs
n	4465	2319	2146	2034	2431
Sensitivity	0.806	0.782	0.830	0.867	0.774
	[0.778,0.834]^a^	[0.740,0.824]	[0.791,0.867]	[0.826,0.906]	[0.737,0.810]
Specificity	0.644	0.668	0.616	0.585	0.702
	[0.621,0.666]	[0.638,0.699]	[0.583,0.648]	[0.553,0.618]	[0.672,0.732]
PPV	0.486	0.477	0.495	0.379	0.586
	[0.459,0.515]	[0.439,0.516]	[0.457,0.534]	[0.342,0.418]	[0.548,0.622]
NPV	0.888	0.888	0.889	0.938	0.851
	[0.871,0.905]	[0.864,0.910]	[0.863,0.913]	[0.918,0.956]	[0.825,0.876]
LR +	2.26	2.36	2.16	2.09	2.60
	[2.11,2.43]	[2.13,2.63]	[1.96,2.38]	[1.91,2.30]	[2.32,2.90]
LR -	0.30	0.33	0.28	0.23	0.32
	[0.26,0.35]	[0.26,0.39]	[0.22,0.34]	[0.16,0.30]	[0.27,0.38]
AUROC^b^	0.725	0.725	0.723	0.726	0.738
	[0.707,0.745]	[0.699,0.751]	[0.698,0.748]	[0.701,0.752]	[0.714,0.762]
Accuracy	0.692	0.700	0.683	0.649	0.727
	[0.674,0.710]	[0.675,0.725]	[0.656,0.709]	[0.621,0.676]	[0.704,0.751]
Overtriage	0.356	0.332	0.384	0.415	0.298
	[0.334,0.378]	[0.304,0.362]	[0.351,0.417]	[0.383,0.446]	[0.269,0.329]
Undertriage	0.194	0.218	0.170	0.133	0.226
	[0.167,0.222]	[0.176,0.261]	[0.133,0.209]	[0.094,0.176]	[0.189,0.263]

*PPV* positive predictive value, *NPV* negative predictive value, *LR* Likelihood ratio, *AUROC* Area under the reciever operating characteristic curve

^a^ Bootstrapped 99% confidence intervals

^b^ Calculated on AUROC curve predefined sensitivity and specificity based on cutoffs in RETTS-A triage levels Red and Orange

**Table 4:** Comparison of outcome measurements between RETTS-a combined Red/Orange level and NEWS medium/high and NEWS medium/high

		RETTS-A	NEWS	NEWS 2^a^
Time-sensitive condition	*n* = 473			
	Se	0.732	0.370	0.345
		[0.679,0.782]^b^	[0.313,0.429]	[0.290,0.402]
	Sp	0.540	0.789	0.833
		[0.519,0.560]	[0.772,0.805]	[0.818,0.848]
	PPV	0.158	0.172	0.197
		[0.139,0.179]	[0.141,0.203]	[0.161,0.232]
	NPV	0.944	0.914	0.915
		[0.932,0.956]	[0.900,0.926]	[0.902,0.927]
	ACC	0.560	0.744	0.781
		[0.541,0.579]	[0.727,0.761]	[0.765,0.797]
	AUROC^c^	0.636	0.579	0.589
		[0.607,0.662]	[0.549,0.609]	[0.560,0.619]
Deviating VS/complication within 48 h	*n* = 455			
	Se	0.908	0.774	0.636
		[0.870,0.941]	[0.723,0.822]	[0.581,0.691]
	Sp	0.558	0.834	0.873
		[0.538,0.578]	[0.818,0.849]	[0.860,0.887]
	PPV	0.189	0.345	0.389
		[0.168,0.211]	[0.307,0.384]	[0.347,0.433]
	NPV	0.982	0.970	0.964
		[0.974,0.989]	[0.962,0.977]	[0.955,0.972]
	ACC	0.594	0.828	0.857
		[0.575,0.613]	[0.813,0.841]	[0.843,0.870]
	AUROC	0.733	0.804	0.792
		[0.713,0.753]	[0.777,0.830]	[0.762,0.820]
Admission	*n* = 2287			
	Se	0.590	0.339	0.301
		[0.564,0.616]	[0.314,0.365]	[0.276,0.327]
	Sp	0.617	0.888	0.935
		[0.590,0.643]	[0.871,0.906]	[0.921,0.948]
	PPV	0.618	0.762	0.829
		[0.590,0.644]	[0.727,0.795]	[0.794,0.862]
	NPV	0.589	0.562	0.560
		[0.562,0.615]	[0.540,0.583]	[0.539,0.581]
	ACC	0.603	0.607	0.610
		[0.585,0.622]	[0.588,0.626]	[0.591,0.629]
	AUROC	0.603	0.614	0.618
		[0.585,0.622]	[0.598,0.629]	[0.604,0.632]
48 h mortality	*n* = 44			
	Se	0.909	0.727	0.727
		[0.795,1.000]	[0.545,0.886]	[0.545,0.886]
	Sp	0.515	0.777	0.819
		[0.496,0.534]	[0.760,0.793]	[0.804,0.834]
	PPV	0.018	0.031	0.039
		[0.011,0.026]	[0.019,0.046]	[0.023,0.057]
	NPV	0.998	0.997	0.997
		[0.996,1.000]	[0.994,0.999]	[0.994,0.999]
	ACC	0.519	0.776	0.818
		[0.499,0.538]	[0.760,0.792]	[0.804,0.833]
	AUROC	0.712	0.752	0.773
		[0.646,0.759]	[0.661,0.831]	[0.681,0.855]
30-day mortality	*n* = 209			
	Se	0.732	0.612	0.536
		[0.651,0.809]	[0.526,0.699]	[0.445,0.627]
	Sp	0.523	0.791	0.831
		[0.503,0.543]	[0.774,0.807]	[0.816,0.846]
	PPV	0.070	0.126	0.135
		[0.056,0.084]	[0.099,0.152]	[0.106,0.166]
	NPV	0.975	0.976	0.973
		[0.967,0.983]	[0.970,0.983]	[0.966,0.980]
	ACC	0.533	0.782	0.817
		[0.513,0.553]	[0.766,0.798]	[0.802,0.832]
	AUROC	0.627	0.702	0.684
		[0.586,0.667]	[0.657,0.746]	[0.639,0.728]

*Se* sensitivity, *Sp* specificity, *PPV* positive predictive value, *NPV* negative predictive value, *AUROC* area under the recieving operator curve

^a^ RETTS-A, NEWS and NEWS2 have been compared with the predefined cut off levels, RETTS-a (Orange/Red), NEWS (Medium/High), NEWS2 (Medium/HIgh)

^b^ Bootstrapped 99% confidence intervals

^c^ AUROC calculations based on predefined cut-off values for each system

## Results

### Patient characteristics and EMS-assigned RETTS-A triage levels

The median age of the 4465 patients included in the study was 69 years and 52% were females. The patients that were triaged to a lower acuity, tended to be older and more frequently, a female. Patients triaged to a high acuity were also dispatched with priority 1 in the majority of cases. Thus, the proportion of patients who were dispatched with priority 1 by the nurse at the scene were red, 77%; orange, 58%; yellow, 39%; and green, 24%. The most common DMI was ‘chest pain’, but with no significant difference between triage groups. The DMI of ‘respiratory difficulties’ was the most frequent (26%) among patients with the highest triage level (red). Being in contact with the EMS during office hours (08:00–16:00) was more common among patients who were triaged to a low acuity, whereas being in contact with the EMS during the night (24:00–08:00) was more common if triaged red (21%) and orange (19%), as compared with yellow and green. Time at the scene was longer for the high-acuity group, compared with the low acuity. For this reason, level red had a median time of 25 min, which was 4 min longer than that in levels green and yellow. The two most common medical history diagnosis groups were ‘diseases of the circulatory system’ and ‘mental and behavioural disorders’. The EMS nurse assessed a higher proportion of patients having abdominal/flank pain with levels yellow and green, compared with level orange/red, whereas, the opposite was found for ‘chest/thoracic pain’, with the highest percentage found in level orange (12%). ‘Chest/thoracic pain’ was more predisposed to high-acuity triage, together with ‘respiratory distress/dyspnoea/breathing difficulties’, which comprised 22% of red triage patients. In the ED, more patients in the high-acuity group were admitted to in-patient care than in the low-acuity group, with the red triage level having the highest frequency (81%). Circulatory and respiratory system-related hospital diagnosis were more common in the high-acuity group, whereas the hospital diagnosis of symptoms, signs and abnormal clinical and laboratory findings were more common in the low-acuity group. All-cause mortality at 7 days, 30 days and 365 days was associated with a higher acuity and thus increased with the higher triage levels (Table 1).

### Probability of outcomes and EMS-assigned RETTS-A triage levels

Red-triaged patients had a three times higher probability of having a time-sensitive condition than the non-red-triaged patients. The occurrence of a deviating VS/complication was 13 times higher while the probability of dying within 48 h was 6 times higher for red-triaged patients. For the lowest triage level (green), there was a low to non-existent risk of developing any of the outcomes. However, there was no significant difference in risk ratios between the yellow and the green group in the occurrence of complications. Yellow- and green-triaged patients had a 79–100% lower risk of death within 48 h. Thirty-one per cent were admitted to in-patient care if triaged to green (Table 2).

### Performance of pre-hospital RETTS-A triage

Overall, patients that were triaged to orange or red had an 81% sensitivity in detecting the predefined reference patient, and a 64% corresponding specificity, which implied an over-triage of 36% and under-triage of 19%. In the patients who were triaged to level green or yellow, the NPV was 89%, whereas the corresponding PPV was 49%. The accuracy and AUROC showed similar results, at 69 and 73%, respectively. Patients over 65 years of age had a lower sensitivity (77%) compared with those under 65 (87%), with a corresponding higher specificity of 70 and 59%, respectively. In the older group, the PPV was significantly higher (59%) than in the younger group (38%). The corresponding NPV was lower for those over 65 years of age, at 85%, compared with the younger group (94%). The diagnostic accuracy was higher in the older group (73%) than in the younger group (65%). The younger group had a higher over-triage (42%) than the older group (30%). The older patients had a higher under-triage (23%) than the younger group (13%) (Table 3).

### Comparison of outcome measurements between RETTS-A, NEWS, and NEWS 2

The sensitivity of detecting a time-sensitive condition was higher with RETTS-A than with both NEWS and NEWS 2 (73, 37, and 35%), whereas the specificity was higher in NEWS 2 (83%) than RETTS (54%). The NPV was higher in RETTS-A (94%) than in both NEWS (91%) and NEWS 2 (92%). The accuracy was higher in NEWS 2 (78%), than in both NEWS (74%) and RETTS-A (56%). We found no significant difference between the three instruments when calculating the AUROC with the specified cut-offs for an emergent patient. In detecting 48-h mortality, NEWS 2 had higher specificity and accuracy (82%) than both NEWS (78%) and RETTS-A (52%). In terms of complications or deviating VS within 48 h, RETTS-A had higher sensitivity (91%) than NEWS (77%) and NEWS 2 (64%), whereas NEWS 2 had higher specificity (87%) than NEWS (83%) and RETTS-A (56%). For hospital admission, the sensitivity with RETTS-A was higher (59%) than with NEWS (34%) and NEWS 2 (30%); however, RETTS-A also had lower specificity and PPV than NEWS and NEWS 2. In predicting 30-day mortality, RETTS-A had a higher sensitivity (73%) than NEWS 2 (54%), albeit no significant difference compared to NEWS. RETTS-A had lower specificity than NEWS and NEWS 2, respectively. Both the PPV and the accuracy were lower in RETTS-A than in NEWS and NEWS 2 (Table 4).

### Agreement between EMS nurse’s field diagnosis and physician’s hospital diagnosis

Of 4465 patients overall, 4168 received diagnosis according to the International Statistical Classification of Diseases and Related Health Problems - Tenth Revision, Swedish edition (ICD-10-SE). Categorising the hospital diagnosis (Table 5), 473 (11%) patients were classified as time-sensitive, 2646 (64%) were classified with a final diagnosis which was not time-sensitive, 808 (19%) were diagnosed with a symptom and 241 patients (6%) were diagnosed with a non-specific assessment. The EMS nurse’s field assessment was appropriate in 82% of the cases. In patients with a defined time-sensitive condition, the EMS nurse’s field assessment was considered appropriate in 395 cases (84%) (Table 5).

**Table 5:** Agreement between the EMS nurse’s field assessment and the final hospital physician diagnosis

	EMS field assessment (*n* = 4168)^a^
**A. A defined final diagnosis classified as a time-sensitive condition**	***n*** **= 473**
1. The field diagnosis is in agreement with the final diagnosis	195 (41.2)
2. The field diagnosis is not in agreement with the final diagnosis	49 (10.4)
3. Typical symptoms related to the final diagnosis	160 (33.8)
4. Atypical symptoms related to the final diagnosis	32 (6.8)
5. More unusual symptoms related to the final diagnosis	8 (1.7)
6. The field assessment as a non-specified organ system	29 (6.1)
**B. A defined final diagnosis not classified as a time-sensitive condition**	***n*** **= 2646**
1. The field diagnosis is in agreement with the final diagnosis	755 (28.5)
2. The field diagnosis is not in agreement with the final diagnosis	308 (11.6)
3. Typical symptoms related to the final diagnosis	1099 (41.5)
4. Atypical symptoms related to the final diagnosis	173 (6.5)
5. More unusual symptoms related to the final diagnosis	104 (3.9)
6. The field assessment as a non-specified organ system	207 (7.8)
**C. The final diagnosis is expressed as a symptom**	***n*** **= 808**
1. The field diagnosis is in agreement with the final symptom	24 (3.0)
2. The field diagnosis is not in agreement with the final symptom	48 (5.9)
3. The field symptom and the final symptom are in agreement	620 (76.7)
4. The field symptom and the final symptom are not in agreement	82 (10.1)
5.The field assessment as a non-specified organ system	34 (4.2)
**D. The final diagnosis is described as a non-specific assessment**	***n*** **= 241**
1. The field diagnosis is in agreement with the symptom	59 (24.5)
2. The field diagnosis is not in agreement with the symptom	42 (17.4)
3. The field symptom is in agreement with the final assessment	84 (34.9)
4. The field symptom and final assessment are not in agreement	20 (8.3)
5. The field assessment is presented as a non-specified organ system	36 (14.9)

^a^ Total number of final hospital diagnoses in 4465 patients

## Discussion

In this study, among the population in contact with the EMS and assessed as needing to be sent to the hospital, the median age was 69 years and 87% had a medical history (circulatory and psychiatric diagnoses were the most common). The three most common field assessments according to RETTS-A were abdominal pain, chest pain, and dyspnoea. Our main findings regarding sensitivity and specificity were similar to those in other studies regarding major triage systems (Manchester triage system [MTS], Emergency severity index [ESI], South African triage scale [SATS]) in the EDs [18–21], albeit with a lower specificity indicating a higher rate of false positives in RETTS-A when used in a pre-hospital context. These systems have been reported in systematic reviews to have moderate to good validity with reasonable performance but high variability and different outcome measures [18, 22, 23]. Clinical competence plays a role in the patients’ assessment, and different triage levels may be reported depending on EMS nurse decisions based on the collected information on patient history and interpretation of the clinical presentation. In a systematic review, Considine and colleagues reported that factual knowledge is more important in triage decisions than triage or emergency nursing experience [24]. Furthermore, the EMS nurse has mostly only one patient to consider at-the-scene, whereas the triage nurse in the ED may be influenced by the current situation and triage in order to solve logistical problems [25]. This suggests that context plays a role. Zachariasse and colleagues reported that other factors influence the performance of triage systems such as infrastructure, nurse experience and epidemiology [22]. In a pre-hospital context, the patient population in contact with the EMS ranges from trivial problems to major traumas and severe medical diseases. This imposes greater demands on a triage system to aid in both these scenarios, however, no uniform guidelines exist within the Swedish ambulance organisations when it comes to referral to lower levels of care leading to local variations. In this study, 31% of the green-triaged patients were hospitalised, indicating that the EMS nurse’s actual competence is important in order to decide which green-triaged patient could remain at the scene and which needed to be transported. Furthermore, it is worth considering that among the EMS population, many patients with chronic diseases may not yield a ‘life-threatening’ triage level, though the patients may still be in need of in-patient care. Therefore, variations in the definition of outcomes for admission, based on severity, or the definition of a reference patient in other studies for example, makes comparisons difficult. In a systematic review of 57 studies of triage systems and their performance, a total of 33 different outcome measurements were used [26]. In order to better compare triage systems, suggestions for a consensus on uniform reporting as in the Utstein style [27], as well as a consensus on what constitutes a time-sensitive condition [28] are required.

Regarding over-triage, there appears to be a consensus that over-triage should be built into the system and perhaps even more so in a pre-hospital context. However, overly extensive over-triage may have implications not only in terms of the unnecessary allocation of resources in the ED but also for the EMS nurse. When the triage system indicates acuity (orange, red), there is no option other than ALS ambulance transportation to the hospital. This may cause an ‘alarm inflation’, together with high levels of over-triage at dispatch (5:1 ratio found in this study) and may induce a lack of trust in the system, with reduced adherence. Adherence to pre-hospital guidelines appears to be influenced by several factors, such as the relationship between patient outcomes and guideline evidence [29]. Pre-hospital over-triage is also associated with increased costs when transporting low-urgency patients to high-resource hospitals when these resources are not needed [30]. This may suggest that using the same triage systems both in the pre-hospital setting and, in the ED, could be favourable. Furthermore, in static triage systems based on expert opinion, when an adverse event occurs, interest often focuses on adjusting the system based on the single adverse event. This have previously been reported in accident investigations where the investigation often stops at the level “preventable causes”, because it is easy to resolve and practical to implement [31]. This may lead to a further increase in over-triage. Regarding under-triage, similar findings relating to under-triage in the ED have been reported in other studies of the MTS, ESI, and SATS (9–25%) [21, 32–35], with higher under-triage in the elderly [35, 36]. This was also found in this study (13.3% ≤ 65 yrs. vs 22.6% >  65 yrs.). This is a concern, as patients in contact with the EMS are often older and the three most frequent hospital diagnoses in the under-triage group were stroke/transient ischaemic attack, sepsis, and myocardial infarction. Patients with these diagnoses were commonly triaged to the RETTS-A category of non-specific complaints. Previous studies have shown that elderly patients with non-specific complaints in the ED have a higher short-term mortality, are to be triaged as less urgent and require resources and hospitalisation to a greater extent than patients with specific complaints [37, 38]. There is also a risk of patients being referred to other levels of care with the aid of a triage system, with older patients being more frequently triaged to non-specific complaints than younger patients [39]. A study of the RETTS-A in the ED reported increased 7-day and 30-day mortality in patients over 60 years of age who were triaged to green level [4]. However, we found that, regardless of age, the risk of death within 48 h was none to very low if triaged to level green or yellow, with a six-fold increase in risk if triaged to level red. This indicates that short-term mortality increases with increasing triage level when triaging with RETTS-A in the EMS and reflects the objective of the system. While many triage systems highlight frail patients, the alternative triage guidelines in trauma outperformed the existing guidelines in the elderly population in the EMS [40]. Furthermore, using the same VS definitions of severity levels regardless of age could miss critically ill elderly patients and thus jeopardise patient safety [41]. This suggests a different set-up regarding triage in the elderly, with specific cut-offs for the ageing patient.

The introduction of NEWS in the EMS, adding all VS to obtain a total score and thereby addressing the severity, may be one way of addressing the problem. In this study, NEWS 2 showed higher accuracy than RETTS-A for the prediction of a time-sensitive condition, 48-h mortality and deviating VS/complications within 48 h. On the other hand, RETTS-A showed greater sensitivity for a time-sensitive condition and deviating VS/complications. Furthermore, RETTS-A showed a higher NPV for time-sensitive conditions than both NEWS and NEWS 2, and combining ESS and VS to yield a triage level is a strength of RETTS-A, but at the expense of an increase in the number of false positives. The sensitivity was relatively low for both NEWS and NEWS 2, which also reflected the accuracy, indicating that NEWS is more capable of ruling out critical conditions but at the expense of more false negatives. However, despite the higher sensitivity, the under-triage of some patients (for example, those with sepsis), may indicate more difficulties when using RETTS-A to identify severe diseases if the symptoms are vague. Examples are proneness to falling (ESS yellow) or a slight deviation in VS, which may yield level yellow for single VS. Whereas, the NEWS, on the other hand, may have better capabilities to detect a deteriorating patient with a score of combined VS. This is also supported by other studies of sepsis, where NEWS was superior to both RETTS-A and qSOFA in the prediction of intensive care and 30-day mortality [42, 43].

Regarding at-the-scene time, the main purpose of the RETTS-A triage system in the ED is to assess patient urgency and time to physician in cases where red-triaged patients are thought to require immediate attention. In the EMS, the nurse is able to intervene at the scene when a condition is identified. The prolonged at-the-scene time at red-triaged level can therefore be explained by the examinations and interventions that were performed. Patients presenting with dyspnoea were common in these cases. In a Danish study on patients with dyspnoea, the 1-day mortality did not increase with a total transport time of > 30 min. However, for cerebrovascular conditions, a total at-the-scene time of > 60 min increased short-term mortality, suggesting faster transportation times in such cases [44]. A short at-the-scene time is essential for several conditions where definitive care is required in the hospital. In clear cases of stroke with the sudden onset of hemiplegia, symptom presentation may not be difficult to assess and manage. However, in the case of the older patient who is experiencing vertigo but has otherwise normal VS and is awake, it may be more complicated due to the vague symptom presentation. This indicates that support from a triage system in the field is valid and may aid in the decision-making process. The performance of triage systems in the EMS may also improve if they are linked to a decision-support system.

The EMS nurses’ field assessments, regardless of RETTS-A triage, were in agreement with the final hospital diagnosis in 82% of all the pre-hospital assessments and in 84% of all time-sensitive conditions. For the assessment of time-sensitive conditions in the field, our findings are similar to those of other studies of sepsis and MI, reporting 78–94% agreement between paramedics and hospital physicians assessments [45–47]. One explanation on the inaccurate field assessments of time-sensitive conditions is the inability to identify the condition as time-sensitive due to the lack of competence and lack of pre-hospital equipment, such as blood tests and instruments, to safely rule out time-sensitive conditions. Several diagnosis groups have been described as being more difficult to differentiate from others, as they have a symptomatic overlap. Such diagnoses include subarachnoid haemorrhage and migraine [48]. The EMS nurse may be influenced by factors that may bias the assessment, such as psychiatric illness; a medical history that was common among the patients in this study. In a study on experienced emergency physicians’ assessments in the ambulance, the agreement with hospital discharge diagnosis was 90%, but it decreased by almost 10% in patients with neurological diseases. The authors conclude that medical history at the scene is essential, together with a thorough patient examination, including laboratory tests such as glucose and electrocardiogram (ECG) recording [49]. In order to aid the EMS nurse in complex clinical decision-making, point-of-care tests and guidelines, together with a triage system that is more adapted for the elderly population, seems reasonable.

### Strengths and limitations

The strength of this study was the relatively large patient cohort that was manually reviewed, together with the prospective aspect involving staff training in the triage system in order to minimise bias in patient records when retrospectively collected. Furthermore, most of the time, the EMS nurse had only one patient to deal with compared with the ED nurse, which may have reduced the risk of triaging on premises other than patient severity. The main limitation is that data collection took place in a single urban setting with short transportation times; thus, generalising the results may be problematic. Moreover, VS were collected from the registry and are reported by the EMS nurse. VS may be measured but never recorded. However, it is unlikely that abnormal VS are not recorded. In cases with a low level of triage, it is more likely that those unrecorded VS fall within the corresponding range of that colour, and MICE imputation therefore appears valid. The triage level may differ between the EMS nurse’s and the ED nurse’s assessments, as has been reported in a Canadian study [50]. However, a divergence in triage level may have several possible reasons and should be seen as continuous care where the patient may deteriorate or improve over time. Furthermore, this study was conducted on a RETTS-A version available in 2016, while RETTS-A is updated annually in order to reduce under-triage, and some ESS codes may have changed since then.

## Conclusions

The median age of the population was 69 years and 87% had a previous medical history. Compared with a predefined reference patient, the sensitivity of RETTS-A was 81% and the specificity was 64%, with over-triage of 36% and under-triage of 19%. An increased risk of an adverse event was identified among the elderly. NEWS and NEWS 2 appeared to have performed better on the outcomes related to VS, mainly due to a higher specificity, while RETTS-A predicted time-sensitive conditions better than NEWS and NEWS 2, and showed a higher proportion of correctly classified low risk triaged patients. Despite that the EMS nurse’s competence play a role in the at-the-scene assessment, over-triage may be unavoidable with the current systems used in the EMS and point-of-care testing and increased medical consultation is proposed. Given the low risk of death in the green triage group, more patients could be diverted to the primary care physicians.

## Supplementary information

**Additional file 1.** Definition of time-sensitive conditions.

## Data Availability

The datasets analysed during the current study are available from the corresponding author in response to a reasonable request.

## Abbreviations

AR: Absolute risk
ALS: Advanced lifesaving
AUROC: Area under the receiver operating characteristic curve
CI: Confidence interval
DMI: Dispatcher medical index
ECG: Electrocardiogram
ED: Emergency department
EMS: Emergency medical services
EMT: Emergency medical technician
ESI: Emergency severity index
ESS: Emergency signs and symptoms
ICD-10-SE: International Statistical Classification of Diseases and Related Health Problems – Tenth Revision, Swedish edition
MTS: Manchester triage system
MAR: Mostly at random
MICE: Multivariate imputation via chained eqs.
MI: Myocardial infarction
NEWS: National early warning score
NPV: Negative predictive value
PPV: Positive predictive value
qSOFA: Quick sequential organ failure assessment score
RETTS-A: Rapid emergency triage and treatment system for adults
RN: Registered nurse
RR: Relative risk
SATS: South African triage scale
VS: Vital signs
