# Supplementary material for: Pre-hospital triage performance and emergency medical services nurse’s field assessment in an unselected patient population attended to by the emergency medical services: a prospective observational study
Source: Scand J Trauma Resusc Emerg Med. 2020 Aug 17;28:81. doi: 10.1186/s13049-020-00766-1 (PMC7430123; doi:10.1186/s13049-020-00766-1)
Supplement: Supplementary file 1 — Additional file 1. Definition of time-sensitive conditions. [file 13049_2020_766_MOESM1_ESM.docx]

| Medical | Traumatic injuries |
| --- | --- |
|  |  |
| Aortic rupture | Cardiac contusion |
| Aortic dissection | Cardiac tamponade |
| Any form of shock | Diaphragm rupture |
| Cardiac arrest | Esophageal rupture |
| Failing heart conducting system | Flail chest |
| Heart failure including pulmonary edema | High energy trauma |
| Intoxication | Massive heamothorax |
| Myocardial infarction | Obstructive airway |
| Pulmonary embolism | Open pneumothorax |
| Septicemia | Pulmonary contusion |
| Tia/Stroke | Tension pneumothorax |
| Unconsciousness | Thoracic aortic rupture/dissection |
| Unstable angina pectoris | Tracheobronchial rupture |

Additional table 1. Definition of time-sensitive conditions for adults

Hagiwara, MA., Nilsson, L., Strömsöe, A., Axelsson, C., Kängström, A. & Herlitz, J. (2016). Patient safety and patient assessment in pre-hospital care: a study protocol. *Scand J Trauma Resusc Emerg Med*. doi: 10.1186/s13049-016-0206-7.

Yamamoto, L. Schoreder, C. Morley, D. & Beliveau, C. (2005). Thoracic trauma: the deadly dozen. Crit Care Nurs Q. 28(1), 22-40.
